# Decreased health-related quality of life in disease-free survivors of differentiated thyroid cancer in Korea

**DOI:** 10.1186/1477-7525-8-101

**Published:** 2010-09-15

**Authors:** Ji In Lee, Soo Hyun Kim, Alice H Tan, Hee Kyung Kim, Hye Won Jang, Kyu Yeon Hur, Jae Hyeon Kim, Kwang-Won Kim, Jae Hoon Chung, Sun Wook Kim

**Affiliations:** 1 Division of Endocrinology and Metabolism, Department of Medicine, Samsung Medical Center, Sungkyunkwan University School of Medicine, Seoul, Korea; 2 Department of Nursing, Inha University, Incheon, Korea

## Abstract

**Background:**

Concern regarding the health-related quality of life (HRQOL) of long-term survivors of thyroid cancer has risen due to the rapid increase in the incidence of thyroid cancer, which generally has an excellent prognosis. The aim of this study was to evaluate the status of HRQOL in disease-free survivors of differentiated thyroid carcinoma (DTC) and to evaluate the important determinants of HRQOL.

**Methods:**

This was a cross-sectional study in which we interviewed consecutive disease-free survivors of DTC. Three different validated questionnaires ("EORTC QLQ-C30" for various functional domains, the "brief fatigue inventory (BFI)" and the "hospital anxiety and depression scale" (HADS)) were used. Data from a large, population based survey of 1,000 people were used as a control.

**Results:**

The response rate for the questionnaires was 78.9% (316/401). Disease-free survivors of DTC showed a decreased HRQOL in all five functional domains (physical, role, cognitive, emotional, and social) on the EORTC QLQ-C30 compared with controls (*P *< 0.01). BFI and HADS-anxiety scores also showed greater distress in disease-free survivors of DTC than in controls (*P *< 0.05). A multiple regression analysis for the determinants of HRQOL showed that the HADS-anxiety, HADS-depression, and BFI scores were the most significant components of decreased HRQOL.

**Conclusions:**

Although disease-free survivors of DTC are expected to have disease-specific survival comparable to the general population, they experience a significantly decreased HRQOL. Anxiety, depression, and fatigue were the major determinants of the decreased HRQOL. Supportive psychological care should be integrated into the management of long-term survivors of DTC.

## Background

The incidence of thyroid cancer is rapidly increasing in Korea and in several parts of the world. Differentiated thyroid carcinoma (DTC), mostly small papillary thyroid carcinomas which show excellent prognosis [1-3], account for the majority of the increased incidence.

Although there are some controversies in the management of DTC (papillary and follicular thyroid carcinoma), primary treatment typically consists of surgery, radioactive iodine (RAI) ablation/treatment, and TSH suppressive therapy with levo-thyroxine (T4). These treatment options are accompanied by various kinds of long-term complications such as voice change after thyroid surgery and xerostomia after high cumulative dose of RAI [4].

Since most patients with DTC become free of disease after the initial treatment, the number of disease-free survivors of thyroid cancer continues to grow. Health-related quality of life (HRQOL) is an important factor in caring for long-term survivors of various types of cancer, and every cancer patient needs and deserves appropriate help from health care providers in order to improve their HRQOL [5].

Despite the expectation of normal life expectancy for most disease-free survivors of DTC, there are concerns about their HRQOL. The results of many published reports however, have been inconsistent. Some studies that describe decreased HRQOL in patients with thyroid cancer have been limited by small sample size [6-8], a lack of comparison with healthy control group [6,7,9], or lack of information regarding specific details about thyroid cancer stage, type of thyroid surgery and radioiodine treatment [9]. Hoftijzer *et al*. reported a decreased HRQOL in 153 cured DTC patients compared with the general population, and the most important independent determinant for better HRQOL was the duration of cure [10]. Contrarily, Peltrari *et al*. found that the overall HRQOL of 341 patients with DTC (stage I, II), whose initial treatment was performed at least five years earlier, was comparable to that of the general population [11]. These previous studies did not address the application of a comprehensive panel of quality of life and mental health instruments to a large population of thyroid cancer survivors of diverse stages by cancer-specific questionnaires.

The aim of this study was to compare the HRQOL for disease-free survivors of DTC with that of the general population using validated questionnaires, and to evaluate the important and manageable determinants, especially mental health instrument, of the HRQOL. We also wanted to see whether the different treatment modalities may affect HRQOL of disease-free survivors of DTC.

## Methods

### Patients

The study involved consecutive disease-free patients with DTC who visited the outpatient clinic of the Thyroid Cancer Center, Samsung Medical Center between July 2008 and October 2008. All patients older that 18 years of age were asked to participate and to complete the written questionnaires by themselves at the outpatient clinic. Inclusion criteria were having undergone thyroid surgery with or without radioiodine therapy, the use of T4 replacement continuously for at least six months, absence of clinical or laboratory evidence of DTC at the time of the study, and no further planned therapy for thyroid cancer except T4 replacement. Exclusion criteria were any other acute or chronic co-morbidity which required medical or surgical treatment and could influence their HRQOL, and the administration of RAI within less than six months either for diagnostic or therapeutic purposes since the recent recovery from hypothyroidism could affect the patient's answers. The most commonly listed medical co-morbidities were: diabetes mellitus, hypertension, coronary artery disease, liver disease, kidney disease, lung disease and psychiatric problem. Patients who had detectable thyroglobulin (Tg) levels during TSH suppression or stimulation were also excluded because these patients had a high likelihood for requiring further treatment, which could be a cause of anxiety. Data on patient age and sex were derived from the medical files: the patients were also asked for additional data on marital status (married vs. not-married), highest level of education achieved (graduated from elementary school, middle school, high school, college or university), employment status (employed vs. not employed), religious status (religious vs. non-religious) and subjective financial status (low, middle, or high economic class) using written questionnaire. Data on disease severity parameters were derived from medical records as follows: histology, disease stage, type of operation, number and cumulative dose of RAI, TSH and free T3 level, and time since remission at the time the questionnaire was administered. This study was approved by the Institutional Review Board of Samsung Medical Center. Written consent was obtained from all participants.

### Controls

Sex- and age- matched control group was adopted from a previously published large-scale epidemiologic study to provide reference data for HRQOL in the general Korean population [12,13]. In summary, 1000 members (F:M = 1:1) of the general population from over 15 sites in Korea were surveyed according to probability-proportional-to-size technique. Eligibility criteria for control included being physically and mentally well enough to fill out a questionnaire of communicate with the interviewer.

### Instruments to Assess Health-related Quality of Life

#### 1. European Organization for Research and Treatment of Cancer Quality of Life Questionnaire Core 30 (EORTC QLQ-C30)

The European Organization for Research and Treatment of Cancer (EORTC) QLQ-C30 was developed in 1993 [14]. It is comprised of 30 cancer-specific questions which are used to assess the HRQOL of cancer patients who participate in clinical trials. It incorporates five functional domains (physical, role, cognitive, emotional, and social), three symptom scales (fatigue, pain, and nausea-vomiting) and a global health/QOL scale. Each of these multiple-item scales is scored from 0 to 100, with a higher score representing better HRQOL. We defined the patients group with a score of 33 or less in the five functional domain and global health/QOL scale as a problematic group according to previous literatures [15,16]. Several single-item symptom measurements are also included in EORTC QLQ-C30 and are used to assess commonly reported problems in cancer patients such as dyspnea, appetite loss, sleep disturbance, constipation, diarrhea, and financial problems. However, only the five functional domains and a global health/QOL scales were used to assess the HRQOL in this study because chemotherapy and conventional radiation therapy are not used to treat patients with DTC as in other cancers. The Korean version (Korean EORTC QLQ-C30) was validated and was demonstrated to have the ability to distinguish the subgroups of patients with different performance and HRQOL [17]. The use of this questionnaire was permitted by the Quality of Life Unit of the EORTC http://www.eortc.be.

#### 2. Brief Fatigue Inventory (BFI)

The BFI was developed for the rapid assessment of fatigue in cancer patients. The BFI consists of nine questions on a single page. Fatigue and its interference in daily living are scored by patients on a numerical scale from 0 to 10 [18]. The global score for the BFI is calculated as the mean value of these nine items. Fatigue severity is then categorized into three groups: a global score of 1-3 is considered mild; a score of 4-6 is moderate; and a score of 7-10 is severe. The Korean version of the BFI (BFI-K) has been validated and has demonstrated reliability as a self-rating instrument used to assess fatigue [19]. The BFI-K was provided by the Pain Research Group of the MD Anderson Cancer Center http://www.mdanderson.org.

#### 3. Hospital Anxiety and Depression (HADS)

The HADS was designed to assess depression and anxiety in a medical or surgical outpatient setting that includes cancer patients [20]. It consists of 14 questions related to the two domains of depression and anxiety, with seven questions focus on depression (HADS-D) and the other seven focus on anxiety (HADS-A). Both the HADS-A and HADS-D are scored from 0 to 21, with higher scores indicating greater distress. A normal value ranges from 0-7, a mild disorder ranges from 8-10, a moderate disorder ranges from 11-14, and a severe disorder ranges from 15-21. The Korean HADS has been developed and validated [21]. A license for the HADS-K was acquired from GL assessment http://www.gl-assessment.co.uk.

### Statistics

The EORTC QLQ-C30 was scored according to the EORTC scoring manual. Incomplete questionnaires were handled as per the developer's recommendations. BFI and HADS questionnaires with missing values were not used. We used descriptive statistics for the socio-demographic and clinico-pathologic features of the subjects. Differences in continuous variables between participants and non-participants for the survey were tested by independent samples *t*-test. Differences between groups in categorical variables were tested by chi-square test and for small cell variables, Fisher`s exact test. The one-sample *t*-test was used to compare the means of each domain of questionnaires between disease-free survivors of DTC and the general population controls. We used an analysis of covariance with a generalized linear model to determine significant differences between the groups according to the mode of treatment of thyroid cancer. Multiple regression analysis was used to evaluate the predictors of HRQOL. The independent variables used to predict each of the EORTC QLQ-C30 domains included demographic features (age at diagnosis, age at evaluation, gender, marital status, level of education, employment, religion, and financial status), clinical parameters (type of operation, cancer stage, TSH level, cumulative RAI dose, and time since remission), BFI scores, and the psychological status of the patient (HADS-D and HADS-A scores). The variables that were *P *< 0.2 in univariate analysis of variance tests or were known to be important determinants that affect the HRQOL in other previously published studies on this topic were included in these multiple regression analysis [6-11,22-26]. *P *values of < 0.05 were considered statistically significant.

## Results

### Recruitment results

**Figure 1:**
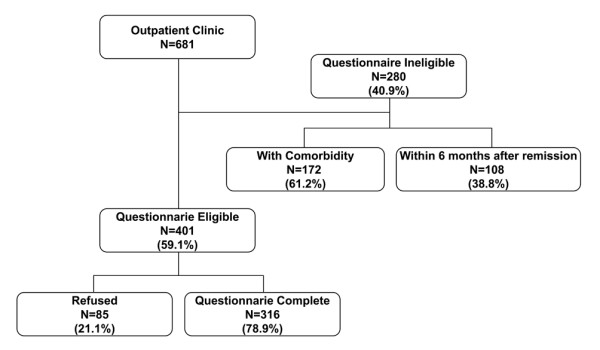
**Recruitment responses to the survey questionnaires**. Four hundred one (59.1%) of 681 potentially disease-free survivors of DTC from participating registries were eligible. Of the 401 disease-free DTC survivors, 316 (78.9%) answered the questionnaires.

We identified 681 consecutive patients at our outpatient clinic who were potentially disease-free survivors of DTC. Two hundred eighty (40.9%) of these patients were excluded either because of a co-morbidity or because it was less than six months after last the administration of RAI. Eighty-five of the 401 (21.1%) disease-free survivors of DTC who were eligible declined participation, and lack of time or inconvenience were the most commonly stated reasons. There were no differences in the demographic and clinico-pathologic characteristics between the participants and the non-participants (Table 1). Three hundred sixteen (78.9%) disease-free survivors of DTC ultimately participated in the study (Figure 1).

**Table 1:** A comparison of demographic and clinicopathologic characteristics between participants and non-participants

Characteristics	Participants (n = 316)	Non-participants (n = 85)	*P *value
Age at diagnosis (yr), mean (SD)	41.3 (9.8)	43.5 (10.2)	0.08
Gender			
Female (%)	287 (90.8)	78 (91.5)	0.84
Marital status (%)			
Married	274/298* (91.9)	55/63^§ ^(87.3)	0.23
Education (%)			
>High school graduate	178/306* (58.2)	34/64^§ ^(53.1)	0.49
Employment status (%)			
Employed	142/307* (46.3)	25/64^§ ^(39.1)	0.29
Religious (%)			
Yes	214/300* (71.3)	42/61^§ ^(68.9)	0.67
Histology (%)			
Papillary carcinoma	308 (97.5)	82 (97.2)	0.89
Follicular carcinoma	8 (2.5)	3 (2.8)	
≥ Stage (AJCC6) III (%)	82 (26.5)	29 (33.8)	0.21
RAI, cumulative dose (mCi), mean (SD)	134.1 (101.1)	120.4 (83.3)	0.29
TSH (uIU/㎖), mean (SD)	0.5 (5.8)	0.2 (0.6)	0.70
Free T3 (pg/㎖), mean (SD)	3.8 (0.9)	3.7 (1.0)	0.36
Time since remission (months), mean (SD)	37.3 (28.8)	38.9 (27.7)	0.70

AJCC6, the American Joint Committee on Cancer 6; RAI, radioactive iodine therapy

* represents number of patients who replied to the specific question.

^§ ^the timing of data collection for marital status, educational level, employment status and religion of non-participants was not at the time of study but at admission for thyroid surgery or radioiodine treatment.

### Patient Characteristics

The baseline clinical characteristics for the participants are summarized in Table 2. Two hundred eighty-seven of the 316 participants were female (90.8%). The mean age (± SD) at the time of diagnosis was 41.2 years (± 9.8). Most of the patients were married (91.9%, 274/298). Two hundred fourteen of the 300 patients (71.3%) were religious, and 93.2% of the patients reported a subjective financial status as middle class or higher. A majority of the participants had papillary thyroid carcinoma (97.5%, 308/316) and eight (2.5%) had follicular thyroid carcinoma. A total thyroidectomy was performed in 89.9% (284/316) of the patients, and 10.1% (32/316) had a subtotal thyroidectomy or lobectomy. Based on TNM staging, 223 (70.6%) of the patients had stage I disease, five (1.6%) had stage II disease, 82 (25.9%) had stage III disease, and six (1.9%) had an unknown stage. The mean TSH level (± SD) at the time of the survey was 0.49 (±5.74) uIU/㎖, free T3 was 3.77 (± 0.93) pg/㎖. The mean time since disease remission (± SD) was 37.3 months (28.8). RAI therapy was performed in 92.0% (291/316) of the patients, with a mean cumulative RAI dose (± SD) of 134.0 (± 101.0) mCi, and the mean number (± SD) of RAI therapy treatments was 2.6 (± 1.4).

**Table 2:** Clinical characteristics of disease-free survivors of DTC

Characteristics	Number of patients (n = 316)	%
Age at diagnosis (yr), mean (SD)	41.2 (9.8)	
Age at evaluation (yr), mean (SD)	46.0 (9.2)	
Gender		
Female	287	90.8
Marital status (n = 298*)		
Married	274	91.9
Education (n = 306*)		
>High school graduate	178	58.2
Employment status (n = 307*)		
Employed	142	46.3
Religious (n = 300*)		
Yes	214	71.3
Subjective financial status (n = 294*)		
≥Middle	274	93.2
Histology		
Papillary carcinoma	308	97.5
Follicular carcinoma	8	2.5
Stage (AJCC6)		
I	223	70.6
II	5	1.6
III	82	25.9
IV	0	0
Unknown	6	1.9
Operation		
Total thyroidectomy	284	89.9
Subtotal/lobectomy	32	10.1
RAI therapy	291	92.0
RAI, cumulative dose (mCi), mean (SD)	134.0 (101.0)	
RAI, total frequency, mean (SD)	2.6 (1.4)	
TSH (uIU/㎖), mean (SD)	0.49 (5.74)	
Free T3 (pg/㎖), mean (SD)	3.77 (0.93)	
Time since remission (months), mean (SD)	37.3 (28.8)	

AJCC6, American Joint Committee on Cancer, sixth edition, stage of disease; RAI, radioactive iodine therapy

* represents number of patients who replied to the specific question.

### Comparison of EORTC QLQ-C30, BFI, and HADS between the Disease-Free Survivors of DTC and the General Population

**Figure 2:**
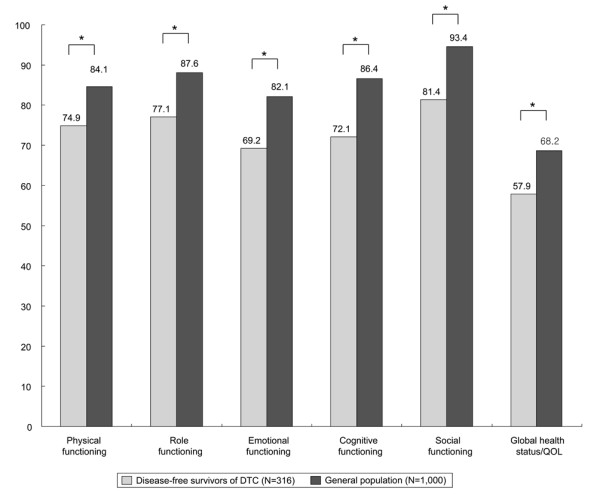
**Comparison of the health-related quality of life between disease-free survivors of differentiated thyroid carcinoma and age/sex-matched controls**. The disease-free survivors of DTC had a statistically significant decrease in all functional domains and global health/QOL scale of EORTC QLQ-C30. *P < 0.05 from one-sample *t*-test

Results from the EORTC QLQ-C30 of the disease-free survivors of DTC and the control group are compared in Figure 2. The disease-free survivors of DTC showed significantly lower scores in all of the functional domains (physical, role, cognitive, emotional, and social), as well as on the global health/QOL scale at the EORTC QLQ-C30 survey (*P *< 0.05). Furthermore, the proportion of problematic groups according to EORTC QLQ-C30 was significantly higher in disease-free survivors of DTC than controls for all functional domains and global health/QOL scale except physical functioning domain (Table 3). HADS-A, HADS-D, and BFI scores for the disease-free survivors of DTC and the control group are shown in Table 4. Disease-free survivors of DTC had greater levels of distress according to the HADS-A and BFI scores. Interestingly, the disease-free DTC patients showed less distress in the HADS-D score compared with the control group.

**Table 3:** Proportion of problematic groups (score≤33 on a scale of 0 to 100) in functional domains and general health/QOL scale of EORTC QLQ-C30

	Disease-free survivors of DTC	Controls	*P *value
Physical functioning	1.6%	1.9%	0.78
Role functioning	8.0%	2.5%	<0.05
Emotional functioning	9.3%	3.2%	<0.05
Cognitive functioning	9.0%	2.0%	<0.05
Social functioning	5.4%	1.9%	<0.05
Global health/QOL scale	17.3%	4.1%	<0.05

**Table 4:** A comparison of the HADS and BFI scores between the disease-free survivors of DTC and the general population

Variable	Disease-free survivors of DTC	Controls
	
	Mean	Mean
HADS		
Anxiety^†^	6.2	5.3
Depression^†^	5.7	6.6
BFI^†^	4.4	3.4

HADS; Hospital Anxiety and Depression Scale. HADS-Anxiety and HADS-Depression are scored from 0 to 21, with higher scores indicating greater distress. Normal (0-7); mild disorder (8-10); moderate disorder (11-14); severe disorder (15-21).

BFI; Brief Fatigue Inventory. global BFI score ranges from 0 to 10, with higher scores indicating greater distress. Mild fatigue (1-3); moderate fatigue (4-6); severe fatigue (7-10). ^†^*P *< 0.05

### HRQOL according to the Mode of Treatment of Thyroid Cancer

The functioning scales for the EORTC QLQ-C30, HADS-A, HADS-D, and BFI did not show any differences between patients who underwent surgery alone and patients who underwent surgery combined with RAI therapy. The cumulative dose of RAI also did not affect the HRQOL in our study group (Table 5).

**Table 5:** HRQOL according to the mode of treatment of thyroid cancer

Variables	Surgery alone (n = 25)	Surgery and RAI < 150 mCi (n = 174)	Surgery and RAI < 150 mCi (n = 117)	
	**mean (SD)**	**mean (SD)**	**mean (SD)**	***P *value**

EORTC QLQ-C30				
Global health status/QOL	57.99 (17.8)	57.90 (21.1)	57.97 (21.8)	NS
Physical functioning	79.93 (13.5)	73.82 (17.1)	75.65 (14.1)	NS
Role functioning	80.56 (20.6)	76.88 (22.41)	76.81 (23.7)	NS
Emotional functioning	67.36 (24.8)	68.66 (23.20)	70.42 (19.6)	NS
Cognitive functioning	76.39 (21.9)	69.85 (21.2)	74.64 (21.7)	NS
Social functioning	82.64 (25.7)	81.50 (20.5)	81.01 (22.1)	NS
HADS				
Anxiety	6.63 (3.9)	6.28 (3.8)	6.15 (3.8)	NS
Depression	6.21 (4.0)	5.80 (3.3)	6.15 (3.8)	NS
BFI, mean score	4.13 (2.0)	4.36 (2.4)	4.53 (2.0)	NS

HRQOL, Health-related quality of life; EORTC QLQ-C30, European Organization for Research and Treatment of Cancer Quality of Life Questionnaire Core 30; QOL, quality of life; HADS, Hospital Anxiety and Depression Scale; BFI, Brief Fatigue Inventory; SD, standard deviation; RAI, radioactive iodine therapy; NS, not significant

Each domain of EORTC QLQ-C30 are calculated and ranges from 0 to 100 and the higher score represents a better level of HRQOL.

HADS; HADS-Anxiety and HADS-Depression are scored from 0 to 21, with higher scores indicating greater distress. Normal (0-7); mild disorder (8-10); moderate disorder (11-14); severe disorder (15-21).

BFI; global BFI score ranges from 0 to 10, with higher scores indicating greater distress. Mild fatigue (1-3); moderate fatigue (4-6); severe fatigue (7-10).

### Determinants for HRQOL

Anxiety, depression and fatigue emerged as the strongest determinants for most of the domains in the EORTC QLQ-C30 in disease-free survivors of DTC according to the multiple regression analysis. Fatigue had negative influence on global health and QOL scale (β = -0.50), physical functioning (β = -0.34), role functioning (β = -0.57), emotional functioning (β = -0.25), cognitive functioning (β = -0.17), and social functioning (β = -0.37). Anxiety had negative influence on physical functioning (β = -1.02), emotional functioning (β = -3.45), cognitive functioning (β = -1.92) and social functioning (β = -1.36). Depression had negative influence on global health and QOL scale (β = -1.82), role functioning (β = -1.41) and cognitive functioning (β= -1.52).

Increasing age at diagnosis (β = -0.27) and female gender (β = -12.11) had a negative influence on the physical functioning domain. Increasing age at evaluation had negative influence on the physical functioning (β = -0.37) and cognitive functioning (β = -0.23) and positive influence on the emotional functioning (β = 0.20).

Patients who were employed at the time of evaluation showed significantly better role functioning (β = 6.66) and social functioning (β = 5.00). The frequency of RAI therapy, cumulative dose of RAI, and level of TSH suppression had no significant impact on the HRQOL of the disease-free survivors of DTC. The regression coefficient of each variables, adjusted R^2 ^and significant *P *values are described in Table 6.

**Table 6:** Determinants for HRQOL using multiple regression including demographic and clinical variables

Variables	Global health status/QOL	Physical functioning	Role functioning	Emotional functioning	Cognitive functioning	Social functioning
Age at diagnosis, years	-	-0.27^a^	-	-0.21^b^	-	-
Age at evaluation, years	-	-0.37 ^a^	-	0.20 ^b^	-0.23 ^b^	
Gender (male = 1,female = 2)	-	-12.11^a^	-	-7.47^b^	-	-
Married (not married = 1, married = 2)	-	-	-	-	-	-
> High school graduate	-	-	-	-	-	-
(≤High school graduate = 1, > High school graduate = 2)	-	-	-	-	-	-
Employed (not employed = 1, employed = 2)	-	-	6.66^a^	-	-	5.00^b^
Religion (non-religious = 1, religious = 2)	-	-	-6.07^b^	-	-5.97^b^	-
≥ Middle class financial status	-	-	-	-	-	-
(<Middle class financial status = 1, ≥ Middle class financial status = 2)	-	-	-	-	-	-
Operation (Total = 1,Subtotal/lobectomy = 2)	-6.57^b^	-	-	-	-	-
Disease stage (stage I,II = 1, stage III,IVa = 2)	-	-	-	-	-4.92^b^	-
TSH (uIU/㎖)	-	-	-	-	-	-
RAI, cumulative dose (mCi)	-	-	-	-	-	-
RAI, total frequency	-	-	-	-	-	-
Time since remission, months	-	-	-	-	-0.11^a^	-
BFI total score	-0.50^a^	-0.34^a^	-0.57^a^	-0.25^a^	-0.17^b^	-0.37^a^
HADS, Anxiety	-	-1.02^a^	-	-3.45^a^	-1.92^a^	-1.36^a^
HADS, Depression	-1.82^a^	-	-1.41^a^	-	-1.52^a^	-

Adjusted R^2^	0.42	0.43	0.39	0.59	0.40	0.25

HRQOL, Health-related quality of life; ^a^*P *< 0.01, ^b^*P *< 0.05; *P *values are from a multiple regression analysis. The numbers in table are regression coefficient (the average increment of HRQOL as the value of each variable one unit).

## Discussion

Our data supports the hypothesis that disease-free survivors with DTC have decreased HRQOL, despite being clinically-free of disease. Important determinants of decreased HRQOL were the patients' subjective fatigue, anxiety, and depression. The modes of treatment (including type of surgery, frequency and cumulative dose of RAI, and level of TSH suppression) did not affect HRQOL in this study population.

Our high response rate of 78.6% resulted in 316 participants, making our study one of the largest to evaluate HRQOL in thyroid cancer patients to date. Furthermore, there were no differences in the demographic and clinico-pathologic characteristics between the participants and the non-participants. Data from a large, population-based, cross-sectional survey of 1,000 Koreans was used as a control in order to limit selection bias.

One limitation of our study was that even though the size of the study population was not small, our study subjects were homogenous in the method of treatment they underwent (total thyroidectomy, 89.9%; at least one dose of radioiodine administration, 92.1%; on T4 suppressive therapy, 97.5%). Also, selection bias may have been introduced due to the socioeconomic characteristics of our institution's geographic location, and the inherent limitation of a single-center study. More than half of the patients (58.2%) had earned at least a college degree, 93.2% of the patients classified themselves as economically middle-class or above, and 90.8% of the patients were women. Although we used general population for controls in the comparisons, we should be cautious in generalizing this study's results to all DTC patients. Further investigation with a larger number of cured DTC patients with more diverse demographic and clinico-pathologic profiles is needed. Furthermore, the relatively short period of follow-up after the determination of cured status (median 2.7 year) precludes any conclusions about the long-term outcomes in these patients, thus follow-up studies should be performed. The other limitation is that we used cancer specific questionnaire "EORTC QLQ-C30" in comparing the general QOL between disease-free survivor and general population. This might have caused some differences from previous reports and future study using questionnaire assessing HRQOL in general population is needed. Lastly, this study was cross-sectional design, which can limit the generalizability of our findings to similar groups of thyroid cancer survivors due to lack of validity of the data collection, lack of initial HRQOL, anxiety, depression and fatigue level and heterogeneous time since the initial thyroid cancer treatment.

We included the EORTC QLQ-C30 in the set of questionnaires in this study. The EORTC QLQ-C30 is one of the most commonly used questionnaires to evaluate HRQOL in various types of cancer. However, to the best of our knowledge, there has been only one report regarding HRQOL using EORTC QLQ-C30 in patients with DTC [27], in which the number of participants was small (n = 62), and the disease status and treatment modalities used for the patients were not specified. In this study we used a group of patients who were all disease-free and included a much larger total number of patients (n = 316).

Hoftijzer *et al*. reported that 153 patients who had been cured of DTC had a decrease in QOL when compared to their healthy controls (n = 113) using multiple questionnaires (SF-36, MFI-20, HADS, SDQ). These decreases were seen in 13 of 16 surveyed areas [10]. They reported that HRQOL may be restored to normal after 12-20 years of follow-up. In our study, even though the time elapsed since cure was relatively shorter (median 2.7 years; range 0.5-13.0) than that of Hoftijzer *et al'*s study (median 6.3 years; range 0.3-41.8), the duration of cure when divided into two groups (<5 years and ≥5) did not influence any aspects of the HRQOL domains of the EORTC QLQ-C30. On the other hand, Pelttari *et al*. used a 15D questionnaire for their study of 341 stage I or II DTC patients who were at least 5 years after cure [11]. They concluded that these cured stage I or II DTC patients showed comparable HRQOL to that of the general Finnish population. In our study, we also incorporated patients with stage III DTC and showed a decreased HRQOL across all stages. Thus, our study corroborates the findings of Hoftijzer *et al*, [10] in showing a decreased HRQOL for cured DTC patients for at least 5-12 years during presumably one of the most active stages of these patients' lives, but deviates from the research of Pelttari *et al*.

Tan *et al*. described that ethnicity may play a role in HRQOL from a study conducted in 152 Singaporeans of diverse ethnicity [22]. Tagay *et al*. also reported that depression and anxiety in patients with DTC are highly correlated with QOL. The most important determinants for depression and anxiety in their study were social support and a sence of coherence; whereas TSH did not show a statistically significant association with depression or anxiety [23]. In addition, it has been reported that patients with head and neck cancers who are more optimistic have a higher HRQOL [28]. Hirsch *et al*. reported that patients with thyroid cancer perceive their illness on a subjective and emotional basis, not on the objective severity of the DTC [29]. So, the influence of different ethnic and cultural background on the perception of illness may have impacted the HRQOL of the cured DTC patients of our study and this may also explain some of the conflicting results in previously reported HRQOL studies. It is possible that in a predominantly ethnically homogeneous country such as South Korea, pervasive perceptions regarding the diagnosis of cancer may profoundly impact how an individual adjusts to DTC. In this regards, the attitude and emotional support by healthcare-provider and family would be of great importance on the HRQOL of long-term survivors of thyroid cancer.

In our study, as in previous studies, treatment modality did not affect HRQOL. The extent of surgery, as in the report by Shah *et al*., did not impact HRQOL, therefore our findings support their statement that HRQOL should not be a factor in the decision of extent of surgery in DTC patients [26] Likewise, we found no relationship between HRQOL and blood TSH level not only as a continuous variable, but also when grouped into suppressed (<0.5 uIU/㎖), normal (0.5-4.5 uIU/㎖) and increased (>4.5 uIU/㎖) categories. A previous report by Eustatia-Rutten *et al*. on a small number of patients who were cured of DTC (n = 24) with > 10 years subclinical hyperthyroidism also showed that HRQOL was preserved except for only minor stable impairment on somatic dysfunction. In their study, restoration of euthyroidism after subclinical hyperthyroidism did not result in consistent improvement of quality of life [25]. In a similar vein, Giusti *et al*. compared 61 DTC patients with a control group consisting of patients on T4 therapy for a non-toxic multi-nodular goiter and found a decreased HRQOL in the DTC patients that was not related to blood TSH levels [7].

In our study, 89.9% of the patients underwent total thyroidectomy and 92% received RAI treatment at least once. The revised American Thyroid Association (ATA) guidelines in 2009 for management of DTC management guidelines recommend near-total or total thyroidectomy without prophylactic central neck dissection, RAI ablation in selected patients, and maintenance of the TSH at or slightly below the lower limit of normal (0.1-0.5 uIU/㎖) for PTC patients at low risk for recurrence [4]. Considering that 93 DTC patients were stratified into the low risk for recurrence category in our study according to the revised ATA guideline, the issue of over-treatment according to older guidelines could be suggested. However, we found no significant differences in HRQOL according to treatment modalities even though the statistical power was weak because most of the patients underwent total thyroidectomy and RAI treatment. The impact of treatment modality needs further assessment with larger number of patients in the future.

We observed that the marital status, education, financial status had little impact on HRQOL. Multivariate analysis revealed that being employed status had a positive influence on role functioning. This reinforces the beneficial effects of the work on their lives or shows that these patients were less affected by the disease and thus still able to continue working.

Lastly, in a study from Germany, Tagay *et al*. showed a decreased HRQOL and a high prevalence of anxiety in DTC patients on T4 suppression therapy, but the prevalence of depression was not increased [23]. Similarly, we found significantly increased HADS-A scores in our subjects compared to that of the general population control. However, the HADS-D scores were significantly lower in the disease-free DTC patients than in the controls. One possible explanation is that TSH suppression in the patient group might be related to the lower HADS-D scores. Further study is required to investigate the relationship between TSH suppression, depression, and anxiety.

## Conclusion

Our study shows that disease-free survivors of DTC patients experience significantly decreased HRQOL in all functional domains of the EORTC QLQ-C30. Anxiety, depression, and fatigue were the major determinants of decreased HRQOL, and further studies are needed to identify their root causes. Anticipatory guidance, psychological supportive care, and improved counseling by physicians and other health care providers who treat disease-free survivors of DTC may lead to improved HRQOL. Studies looking at effective management strategies to ameliorate psychologic disturbances in these patients are also warranted.

## Competing interests

The authors declare that they have no competing interests.

## Authors' contributions

JIL contributed the study design, data collection, statistical analysis, interpretation of data and draft of the paper and revision of the manuscript. SWK contributed to the study design, interpretation of data, draft of the paper and revision of the manuscript. SHK contributed to data analysis and interpretation of data. AHT contributed to the draft and revision of the manuscript. HKK, HWJ, KYH, JHK contributed to data collection and interpretation of data. KWK and JHC supervised execution of the study. All authors read and approved the final manuscript.

## References

[B1] NationalCancer Center of Korea: Cancer Registry and Statistics between 2003-2005 2008 National Cancer Centre http://www.ncc.re.kr

[B2] Leenhardt L Grosclaude P Cherie-Challine L Increased incidence of thyroid carcinoma in france: a true epidemic or thyroid nodule management effects? Report from the French Thyroid Cancer Committee Thyroid 2004 14 12 1056 1060 10.1089/thy.2004.14.1056 15650358

[B3] Davies L Welch HG Increasing incidence of thyroid cancer in the United States, 1973-2002 Jama 2006 295 18 2164 2167 10.1001/jama.295.18.2164 16684987

[B4] Cooper DS Doherty GM Haugen BR Kloos RT Lee SL Mandel SJ Mazzaferri EL McIver B Pacini F Schlumberger M Sherman SI Steward DL Tuttle RM Revised American Thyroid Association management guidelines for patients with thyroid nodules and differentiated thyroid cancer Thyroid 2009 19 11 1167 1214 10.1089/thy.2009.0110 19860577

[B5] Bottomley A Aaronson NK International perspective on health-related quality-of-life research in cancer clinical trials: the European Organisation for Research and Treatment of Cancer experience J Clin Oncol 2007 25 32 5082 5086 10.1200/JCO.2007.11.3183 17991925

[B6] Botella-Carretero JI Galan JM Caballero C Sancho J Escobar-Morreale HF Quality of life and psychometric functionality in patients with differentiated thyroid carcinoma Endocr Relat Cancer 2003 10 4 601 610 10.1677/erc.0.0100601 14713270

[B7] Giusti M Sibilla F Cappi C Dellepiane M Tombesi F Ceresola E Augeri C Rasore E Minuto F A case-controlled study on the quality of life in a cohort of patients with history of differentiated thyroid carcinoma J Endocrinol Invest 2005 28 7 599 608 16218042 10.1007/BF03347258

[B8] Tagay S Herpertz S Langkafel M Erim Y Bockisch A Senf W Gorges R Health-related Quality of Life, depression and anxiety in thyroid cancer patients Qual Life Res 2006 15 4 695 703 10.1007/s11136-005-3689-7 16688502

[B9] Schultz PN Stava C Vassilopoulou-Sellin R Health profiles and quality of life of 518 survivors of thyroid cancer Head Neck 2003 25 5 349 356 10.1002/hed.10217 12692870

[B10] Hoftijzer HC Heemstra KA Corssmit EP van der Klaauw AA Romijn JA Smit JW Quality of life in cured patients with differentiated thyroid carcinoma J Clin Endocrinol Metab 2008 93 1 200 203 10.1210/jc.2007-1203 17956954

[B11] Pelttari H Sintonen H Schalin-Jantti C Valimaki MJ Health-related quality of life in long-term follow-up of patients with cured TNM Stage I or II differentiated thyroid carcinoma Clin Endocrinol (Oxf) 2009 70 3 493 497 10.1111/j.1365-2265.2008.03366.x 18681857

[B12] Yun YH Kim SH Lee KM Park SM Kim YM Age, sex, and comorbidities were considered in comparing reference data for health-related quality of life in the general and cancer populations J Clin Epidemiol 2007 60 11 1164 1175 10.1016/j.jclinepi.2006.12.014 17938059

[B13] Yun YH Lee MK Chun HN Lee YM Park SM Mendoza TR Wang XS Cleeland CS Fatigue in the general Korean population: application and normative data of the Brief Fatigue Inventory J Pain Symptom Manage 2008 36 3 259 267 10.1016/j.jpainsymman.2007.10.016 18411013

[B14] Aaronson NK Ahmedzai S Bergman B Bullinger M Cull A Duez NJ Filiberti A Flechtner H Fleishman SB de Haes JC Kaasa S Klee M Osoba D Razavi D Rofe PB Schraub S Sneeuw K Sullivan M Takeda F The European Organization for Research and Treatment of Cancer QLQ-C30: a quality-of-life instrument for use in international clinical trials in oncology J Natl Cancer Inst 1993 85 5 365 376 10.1093/jnci/85.5.365 8433390

[B15] Hjermstad MJ Fayers PM Bjordal K Kaasa S Health-related quality of life in the general Norwegian population assessed by the European Organization for Research and Treatment of Cancer Core Quality-of-Life Questionnaire: the QLQ = C30 (+ 3) J Clin Oncol 1998 16 3 1188 1196 9508207 10.1200/JCO.1998.16.3.1188

[B16] Fayers PM Interpreting quality of life data: population-based reference data for the EORTC QLQ-C30 Eur J Cancer 2001 37 11 1331 1334 10.1016/S0959-8049(01)00127-7 11435060

[B17] Yun YH Park YS Lee ES Bang SM Heo DS Park SY You CH West K Validation of the Korean version of the EORTC QLQ-C30 Qual Life Res 2004 13 4 863 868 10.1023/B:QURE.0000021692.81214.70 15129896

[B18] Mendoza TR Wang XS Cleeland CS Morrissey M Johnson BA Wendt JK Huber SL The rapid assessment of fatigue severity in cancer patients: use of the Brief Fatigue Inventory Cancer 1999 85 5 1186 1196 10.1002/(SICI)1097-0142(19990301)85:5<1186::AID-CNCR24>3.0.CO;2-N 10091805

[B19] Yun YH Wang XS Lee JS Roh JW Lee CG Lee WS Lee KS Bang SM Mendoza TR Cleeland CS Validation study of the korean version of the brief fatigue inventory J Pain Symptom Manage 2005 29 2 165 172 10.1016/j.jpainsymman.2004.04.013 15733808

[B20] Zigmond AS Snaith RP The hospital anxiety and depression scale Acta Psychiatr Scand 1983 67 6 361 370 10.1111/j.1600-0447.1983.tb09716.x 6880820

[B21] Oh SMMK Park D A study on the standardization of the hospital anxiety and depression scale for Koreans - A comparison of normal, depressed and anxious groups -J Korean Neuropsychiatr Assoc 1999 38 2 289 296

[B22] Tan LG Nan L Thumboo J Sundram F Tan LK Health-related quality of life in thyroid cancer survivors Laryngoscope 2007 117 3 507 510 10.1097/MLG.0b013e31802e3739 17334313

[B23] Tagay S Herpertz S Langkafel M Erim Y Freudenberg L Schopper N Bockisch A Senf W Gorges R Health-related quality of life, anxiety and depression in thyroid cancer patients under short-term hypothyroidism and TSH-suppressive levothyroxine treatment Eur J Endocrinol 2005 153 6 755 763 10.1530/eje.1.02047 16322380

[B24] Tagay S Senf W Schopper N Mewes R Bockisch A Gorges R [Protective factors for anxiety and depression in thyroid cancer patients] Z Psychosom Med Psychother 2007 53 1 62 74 17311732 10.13109/zptm.2007.53.1.62

[B25] Eustatia-Rutten CF Corssmit EP Pereira AM Frolich M Bax JJ Romijn JA Smit JW Quality of life in longterm exogenous subclinical hyperthyroidism and the effects of restoration of euthyroidism, a randomized controlled trial Clin Endocrinol (Oxf) 2006 64 3 284 291 10.1111/j.1365-2265.2006.02458.x 16487438

[B26] Shah MD Witterick IJ Eski SJ Pinto R Freeman JL Quality of life in patients undergoing thyroid surgery J Otolaryngol 2006 35 4 209 215 10.2310/7070.2006.0011 17176794

[B27] Roberts KJ Lepore SJ Urken ML Quality of life after thyroid cancer: an assessment of patient needs and preferences for information and support J Cancer Educ 2008 23 3 186 191 10.1080/08858190802247762 18709591

[B28] Kung S Rummans TA Colligan RC Clark MM Sloan JA Novotny PJ Huntington JL Association of optimism-pessimism with quality of life in patients with head and neck and thyroid cancers Mayo Clin Proc 2006 81 12 1545 1552 10.4065/81.12.1545 17165633

[B29] Hirsch D Ginat M Levy S Benbassat C Weinstein R Tsvetov G Singer J Shraga-Slutzky I Grozinski-Glasberg S Mansiterski Y Shimon I Reicher-Atir R Illness perception in patients with differentiated epithelial cell thyroid cancer Thyroid 2009 19 5 459 465 10.1089/thy.2008.0360 19415995

